# Survival Distinctions for Cases Representing Immunologically Cold Tumors via Intrinsic Disorder Assessments for Blood-Sourced TRB Variable Regions

**DOI:** 10.3390/ijms252111691

**Published:** 2024-10-30

**Authors:** Arpan Sahoo, Etienne C. Gozlan, Joanna J. Song, George Angelakakis, Michelle Yeagley, Boris I. Chobrutskiy, Taha I. Huda, George Blanck

**Affiliations:** 1 Department of Molecular Medicine, Morsani College of Medicine, University of South Florida, Tampa, FL 33612, USA; asahoo@usf.edu (A.S.); thuda@usf.edu (T.I.H.); 2 University of Pittsburgh Medical Center, Pittsburgh, PA 15261, USA; 3 Department of Internal Medicine, Oregon Health and Science University Hospital, Portland, OR 97239, USA; 4 Department of Immunology, H. Lee Moffitt Cancer Center and Research Institute, Tampa, FL 33612, USA

**Keywords:** TRB variable regions, uveal melanoma, intrinsic disorder, survival probability distinctions, neuroblastoma

## Abstract

T cell receptor beta (TRB) sequences were recovered from the Cancer Genome Atlas Uveal Melanoma blood exome files. Intrinsic disorder scores for amino acid (AA) sequences of the entire TRB variable region were obtained and evaluated as potentially representative of overall survival (OS) distinctions, i.e., for cases representing the upper and lower 50th percentiles for intrinsic disorder scores. Analyses using four intrinsic disorder assessment tools indicated that a lower intrinsic disorder of the blood-sourced TRB variable regions, including continuous AA sequences of the V-gene segment, the complementarity-determining region-3, and the J-gene segment, was associated with a better OS probability (with log-rank *p*-values ranging from 0.002 to 0.014). We further determined that intrinsic disorder assessments could be used for OS stratification for a second, immunologically cold cancer: *MYCN* amplified neuroblastoma. Thus, intrinsic disorder assessments of blood-sourced, full TRB variable regions may provide a novel patient stratification approach for patients with immunologically cold cancers.

## 1. Introduction

Uveal melanoma (UVM), while relatively rare, is the most common primary intraocular tumor in adults. The tumor originates from melanocytes in the uvea and often resides in the choroid, a highly vascularized tissue that provides a robust blood source to the retina [1]. Importantly, the choroid is one of the most vascularized tissues in the human body [2], supporting tumor growth and providing a route for metastasis, specifically to the liver in the case of UVM [3].

Primary UVM has classically been treated by enucleation, tumor resection, or radiation therapy, while metastatic UVM has required resection or ablation of metastatic sites in addition to delivery of medication through the hepatic artery (e.g., hepatic artery infusion chemotherapy, immune-embolization with granulocyte-macrophage colony-stimulating factor). Systemic chemotherapy and immune checkpoint inhibitors (e.g., anti-CTLA4 and anti-PD1 agents) have had limited efficacy in treating metastatic UVM [4,5,6], in contrast to cutaneous melanoma, where immune checkpoint inhibitors (ICIs) are an important option for treatment. The lack of effectiveness of ICIs is likely due to the fact that UVM is, at least comparatively, an immunologically cold tumor, as the eye and the primary site of metastasis, the liver, are considered to be immunologically privileged sites [6].

However, increasing evidence suggests that there may be a minimal or underappreciated ocular immune response that warrants further investigation. For example, a single-cell RNA-seq analysis revealed clonally expanded T cells in UVM samples and identified LAG3, rather than PD-1 or CTLA4, as a potential target for immune checkpoint inhibition [7]. A recently published phase 2 trial of tebentafusp, a soluble T cell receptor bispecific, i.e., specific for a peptide-HLA complex, and the T cell expressed CD3, showed a marked improvement in overall survival (OS) rate, from 37% to 62% for metastatic UVM [8]. Also, a phase 2 trial of relatlimab, an anti-LAG3 antibody, in combination with nivolumab, an anti-PD1 antibody, showed a 47.7% progression-free survival rate at 1 year compared to 36.0% in patients treated with nivolumab alone [9]. These findings suggest that an adaptive immune response can be mounted against UVM and have supported the goal of better understanding the correlation of T cell receptor chemical features with UVM OS.

To gain further insight into the chemical features related to antigen recognition and binding, we investigated the intrinsic disorder of the TCR beta chain (TRB) variable region. Intriguingly, unstable conformations, as would be revealed by intrinsic disorder assessments, serve to provide an advantage related to the ability of CLIP to bind multiple MHC class II variations [10]. Furthermore, many intrinsically disordered proteins undergo a disorder-to-order transition upon interaction with a globular molecular binding partner. The potential biological value of having a disorder-to-order transition includes conformational flexibility as a way to bind multiple different targets, the ability to resist factors like extreme pH that would denature ordered proteins, and a reduced half-life to facilitate rapid turnover for regulatory molecules [11,12].

In this report, we evaluated full TRB variable region amino acid (AA) sequences recovered from UVM patients’ blood specimens, with a focus on assessing the association between intrinsic disorder of these sequences and OS probability. We also report survival distinctions based on intrinsic disorder of blood-sourced TRB sequences for *MYCN* amplified neuroblastoma, another tumor considered to be immunologically cold [13].

## 2. Results

### 2.1. OS Probability Distinctions for UVM, Based on Assessments of Intrinsic Disorder for Blood-Sourced TRB V-CDR3-J AA Sequences

**Figure 1:**
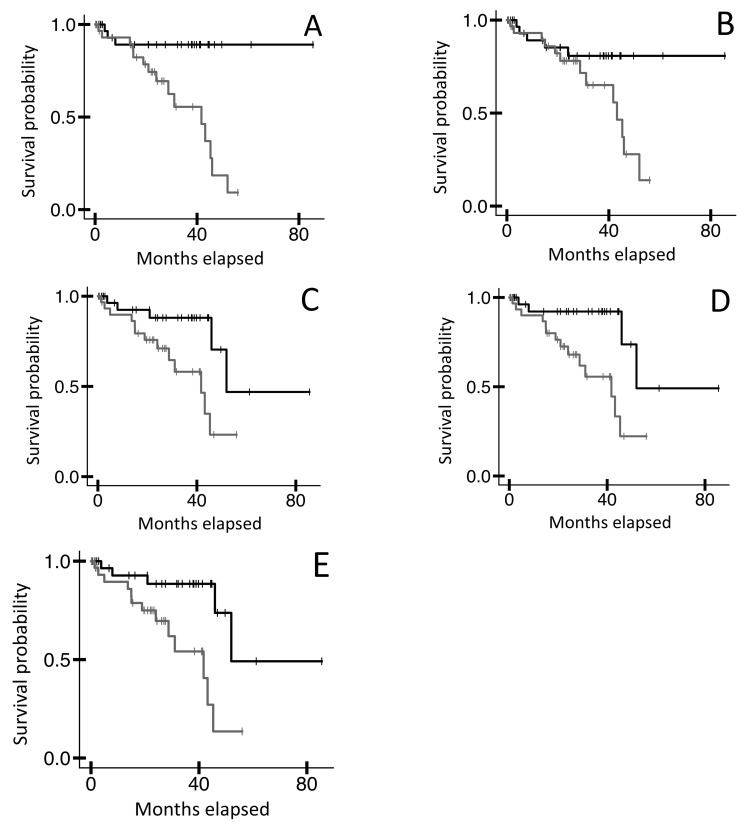
Kaplan–Meier (KM) overall survival (OS) analyses of TCGA-UVM case IDs based on intrinsic disorder assessments as applied to TRB V-CDR3-J AA sequences from blood sample WXS files. (**A**) Case IDs representing the bottom 50% of VSL2 scores (black line, n = 32, median OS N/A) versus the top 50% (grey line, n = 32, median OS 41.7 months). Log-rank *p*-value = 0.002. (**B**) Case IDs representing the bottom 50% of VL3 scores (black line, n = 32, median OS N/A) versus the top 50% (grey line, n = 32, median OS 43.2 months). Log-rank *p*-value = 0.066. (**C**) Case IDs representing the bottom 50% of IUPred2 short scores (black line, n = 32, median OS 52.0 months) versus the top 50% (grey line, n = 32, median OS 41.7 months). Log-rank *p*-value = 0.014. (**D**) Case IDs representing the bottom 50% of IUPred2 long scores (black line, n = 32, median OS 52.0 months) versus the top 50% (grey line, n = 32, median OS 41.7 months). Log-rank *p*-value = 0.004. (**E**) Case IDs representing the bottom 50% of ANCHOR2 scores (black line, n = 32, median OS 52.0 months) versus the top 50% (grey line, n = 32, median OS 41.7 months). Log-rank *p*-value = 0.003.

To assess intrinsic disorder for TRB V-CDR3-J AA sequences from blood specimens of UVM cases, we first mined TRB recombination reads from TCGA-UVM blood WXS files (Section 4; Table S1). The recombination reads were each defined by a validated V and J sequence, as previously described [14], and required an intervening, productive CDR3, i.e., a CDR3 AA sequence with no frameshifts or stop codons. For each recovered TRB recombination read, once the V, CDR3, and J were identified, the appropriate V and J AA sequences were integrated into the CDR3 AA sequence to produce a full-length V-CDR3-J AA sequence representing the full TRB variable region. (See Section 4 for further details.) We then employed multiple computational models, termed VSL2, VL3, IUPred2 short, IUPred2 long, and ANCHOR2, to identify regions of intrinsic disorder in the V-CDR3-J AA sequences (Section 4). Finally, we conducted an OS analysis based on each set of scores resulting from those five intrinsic disorder assessment tools. Results indicated that UVM cases represented by VSL2 scores in the lower 50th percentile (corresponding to a lower likelihood of intrinsic disorder) had a higher OS probability than cases represented by the upper 50th percentile (Figure 1A, n = 64, log-rank *p*-value = 0.002). This distinction was also observed with lower VL3, IUPred2 short, IUPred2 long, and ANCHOR2 scores, though the distinction with the VL3 approach represented only a trend (Figure 1B–E, log-rank *p*-values = 0.066, 0.014, 0.004, 0.003, respectively).

### 2.2. OS Probability Distinctions for UVM, Based on Physico-Chemical Parameters of Blood-Sourced TRB CDR3 AA Sequences

**Figure 2:**
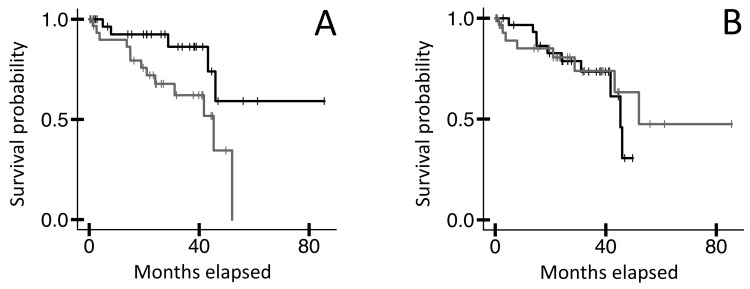
KM OS analyses of TCGA-UVM case IDs based on physico-chemical parameters of TRB CDR3 AA sequences (Table S5) from blood sample WXS files. (**A**) Case IDs representing the bottom 50% of the proportion of disorder-promoting residues (black line, n = 32, median OS N/A months) versus the top 50% (grey line, n = 32, median OS 45.3 months). Log-rank *p*-value = 0.022. (**B**) Case IDs representing the bottom 50% of the proportion of helix-promoting residues (black line, n = 32, median OS 45.3 months) versus the top 50% (grey line, n = 32, median OS 52.0 months). Log-rank *p*-value = 0.631. Similarly to the proportion of helix-promoting residues, two other physico-chemical parameters, namely the proportion of sheet-promoting residues and turn-promoting residues, did not reflect significant OS distinctions.

Because the CDR3, a subset of the variable region, typically represents the most important part of the adaptive IR for antigen binding, we made intrinsic disorder assessments for the CDR3 using parameters effective for the smaller CDR3 AA length. Note, the intrinsic disorder assessment tools used above are not functional for the shorter CDR3 AA lengths. Thus, we instead assessed the following four parameters for the TRB CDR3 AA sequences represented by TRB recombination reads from the blood WXS files in the TCGA-UVM dataset: proportion of (a) disorder-promoting AA residues; (b) α-helix promoting residues; (c) β-turn promoting residues; and (d) β-sheet promoting residues (Section 4). We then assessed potential OS probability distinctions represented by these four parameters. Results indicated that UVM cases represented by the lower 50th percentile of (a) disorder-promoting AA residues in the TRB CDR3s had a higher OS probability than cases represented by the upper 50th percentile (Figure 2A, n = 64, log-rank *p*-value = 0.022). However, the parameters (b) α-helix promoting residues, (c) β-turn promoting residues, and (d) β-sheet promoting residues, which represent structural stability indicators as opposed to disorder indicators, did not reflect any OS probability distinctions (for example, Figure 2B, log-rank *p*-value = 0.631).

### 2.3. An OS Probability Distinction for MCYN Amplified NBL, Based on ANCHOR2 Assessment of Intrinsic Disorder for Blood-Sourced TRB V-CDR3-J AA Sequences

**Figure 3:**
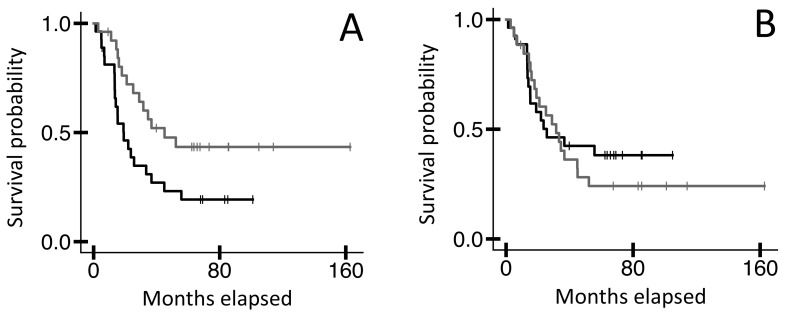
KM OS analyses of TARGET-NBL case IDs based on intrinsic disorder assessments as applied to TRB V-CDR3-J AA sequences from blood sample WXS files. (**A**) Case IDs representing the bottom 50% of ANCHOR2 scores (black line, n = 27, median OS 19.1 months) versus the top 50% (grey line, n = 26, median OS 45.0 months). Log-rank *p*-value = 0.024. (**B**) Case IDs representing the bottom 50% of VSL2 scores (black line, n = 27, median OS 25.6 months) versus the top 50% (grey line, n = 26, median OS 31.5 months). Log-rank *p*-value = 0.600. Like VSL2, the other intrinsic disorder assessments, namely VL3, IUPred2 short, and IUPred2 long, did not reflect significant OS distinctions.

To further explore the value of assessing intrinsic disorder for the entire TRB variable region, we aimed to identify OS distinctions for another (relatively) non-immunogenic cancer type: MYCN amplified neuroblastoma [15,16]. We recovered TRB recombination reads from the blood WXS files in the TARGET-NBL dataset and established the full-length V-CDR3-J AA sequences represented by those TRB recombination reads as described above (Section 4). Then, using the intrinsic disorder assessment tools, we evaluated those V-CDR3-J AA sequences. Results indicated that MCYN amplified NBL cases represented by ANCHOR2 scores in the upper 50^th^ percentile had a higher OS probability compared to those in the lower 50^th^ percentile (Figure 3A, n = 53, log-rank *p*-value = 0.024). However, the VSL2, VL3, IUPred2 short, and IUPred2 long approaches and scores did not reflect any OS probability distinctions (for example, Figure 3B, log-rank *p*-value = 0.600).

### 2.4. Multivariate Analysis

Given the statistically significant OS distinctions between the upper and lower 50th percentiles for the different intrinsic disorder assessments reported above, we aimed to account for potentially confounding clinical variables. For UVM, survival distinctions based on the VSL2, IUPred2 short, IUPred2 long, and ANCHOR2 approaches maintained their statistical significance; additionally, the survival distinction based on the proportion of disorder-promoting residues maintained its statistical significance (Table 1). For *MYCN* amplified NBL, the survival distinction based on the ANCHOR2 approach maintained its statistical significance (Table 2).

## 3. Discussion

Establishing survival distinctions by assessment of the patient’s adaptive immune receptors (IRs) has been difficult for UVM, given the disease’s low immunogenicity. We hypothesized that the application of intrinsic disorder assessment tools, which integrate an array of features ranging from charge-hydrophobicity ratio to intra-protein interaction energies and which can be applied over relatively long polypeptide lengths, would provide an opportunity for greater specificity and power of assessments. Results reported here indicate a significant association between low intrinsic disorder of the blood-sourced TRB variable region and greater overall survival (OS) for UVM patients. In fact, these findings are consistent across various models of intrinsic disorder; we found that low VSL2, IUPred2 short, IUPred2 long, and ANCHOR2 scores were all associated with a greater OS probability for UVM. These results encouraged us to also exploit blood-sourced TRB V-CDR3-Js for the identification of a survival distinction for *MYCN* amplified NBL, another immunologically cold tumor.

These full-length TRB variable region assessment approaches are in contrast to related approaches that have relied on the CDR3 alone. As far as the authors are aware, in all previous attempts to establish survival probability distinctions based on TCR chemistries or chemical fingerprints, the assessments were limited to the CDR3 AA sequences, based on the understanding that the CDR3 AA sequences play a major role in antigen binding. However, the relatively short AA sequence of the CDR3 places an upper limit on specificity and gradations of distinctions in comparison to assessing the entire TRB variable region. Although the CDR3 AA sequence has a relatively unique importance in antigen contacts, it is obvious that other chemical features of the variable region also contribute to the specificity of antigen binding, even if indirectly. Thus, in sum, the results above indicate that a finer and more comprehensive assessment of adaptive IR chemistries (i.e., analysis of the full variable region) may provide opportunities to stratify patients when the immune response is comparatively low. And, again, as far as the authors are aware, this is the first time the survival of UVM cases has been distinguished based on TRB variable region chemical features.

More specifically, in the case of UVM, cases represented by lower TRB intrinsic disorder for the V-CDR3-J region represented better OS probabilities. This is somewhat in contrast to the results obtained with *MYCN* amplified NBL; the *MYCN* amplified NBL cases represented by higher ANCHOR2 scores exhibited greater OS. Note, however, that the ANCHOR2 assessment tool specifically identifies disordered binding sites within proteins that undergo a disorder-to-order transition upon interaction with a hypothetical globular binding partner. In other words, higher ANCHOR2 scores indicate the presence of context-dependent disorder, not just strict disorder. This approach differs from the other intrinsic disorder assessment tools, which more generally detect intrinsically disordered regions without regard for such context-dependent transitions. While there are no data that point to specific antigens, and certainly no data that directly connects the status of blood-sourced TRB polypeptides to an immune response against these two cancers, overall the intrinsic disorder approaches that provided the OS distinctions in this report raise the question of whether there is a specific antigen, or a relatively small set of antigens, that is the target of the TRB variable regions associated with better OS. In the case of UVM, the lower intrinsic disorder would imply rigidity and higher specificity in TRB binding to the antigen. In the case of NBL, the ANCHOR results imply the likelihood of a specific binding partner that facilitates a disorder-to-order transition for the TRB before binding the antigen.

The work reported here has several limitations. First, we analyzed only TRB recombination reads, and although TRB is generally considered more important for directly contacting antigens, TRA chemistry would obviously be important for the function of the entire T cell receptor. Second, we were unable to analyze TRB recombination reads from primary tumor specimens due to extremely low sample sizes and low immune infiltrate. Blood-sourced results are limited to a general picture of the patient’s adaptive immune status, though they may have value as a non-invasive prognostic biomarker, depending, of course, on follow-up studies and verifications of results reported here. Third, while TRB chemical fingerprints may indeed lead to useful patient stratification biomarkers as well as point in the direction of more focused research involving TRB-antigen contacts, a full understanding of TRB-tumor antigen interaction would require not only in vitro studies but also high-resolution computational assessments, as would be provided by molecular dynamics or cyroEM approaches. However, it is important to keep in mind that our results, which are facilitated by large patient datasets and informed by survival outcomes, provide a very credible foundation for further investment into the role of the T cell receptor in regulating two immunologically cold tumors: UVM and *MYCN* amplified NBL. As noted in the Seciton 1, this is consistent with recent, specific attempts to revisit the idea of immunotherapy for UVM. And as for NBL, treatment options have recently expanded to include immunotherapy, such as anti-GD2 monoclonal antibodies as well as CAR-T cell therapy [17], the latter of which specifically motivates research to also explore TRB intrinsic disorder in NBL.

## 4. Materials and Methods

### 4.1. Recovery of TRB Recombination Reads from Uveal Melanoma and Neuroblastoma Datasets

We used previously described methods to recover TRB V(D)J recombination reads from whole exome sequencing (WXS) files [14,18,19]. Access to uveal melanoma (UVM) and neuroblastoma (NBL) WXS files from The Cancer Genome Atlas (TCGA, phs000178) dataset and Therapeutically Applicable Research to Generate Effective Treatments (TARGET, phs000218) dataset was via database of genotypes and phenotypes (dbGaP) project approval numbers 6300 and 16,405, respectively. The latest version of the software for mining adaptive immune receptor (IR) recombination reads is publicly available at https://github.com/arpansahoo/vdj (accessed on 23 September 2024). Complete collections of the TRB recombination read data, for both UVM and NBL, are in the Supplementary Materials Tables S1 and S2.

### 4.2. Determination of AA Sequences Representing the Full-Length TRB Variable Region from Recovered TRB Recombination Reads

We identified the V-gene segment, the J-gene segment, and the complementarity-determining region-3 (CDR3) in each TRB recombination read using the software indicated above and following established methods [14]. The CDR3 AA sequences were translated from the recovered TRB recombination reads, whereas the V- and J-gene segment AA sequences were obtained from https://imgt.org/ [20]. To represent the entire TRB variable region, we subsequently extended the CDR3 AA sequences into full-length V-CDR3-J AA sequences. This was performed programmatically through a string alignment procedure between the CDR3 AA sequence and its associated V and J AA sequences. We first integrated each CDR3 AA sequence with its associated V-gene segment AA sequence (i.e., the V segment as identified by the 5′ end of the sequencing read to just past the first few CDR3 nucleotides). To accomplish this, we defined the longest common substring (LCS) between the CDR3 and V AA sequences, i.e., the AAs shared by the indicated V-gene segment and the first few AAs of the CDR3. (Note, the LCS had to start with the conserved cysteine from the V-gene segment). Then, the LCS and any following residues were removed from the V-gene segment AA sequence, and the full CDR3 AA sequence, including the LCS, was concatenated to the end to build the full “V-CDR3” AA sequence. Next, we integrated the “V-CDR3” sequence with its associated J-gene segment AA sequence, also identified by the sequencing read. AA residues in the J sequence preceding the phenylalanine-glycine doublet motif were removed, and the remaining J sequence was concatenated to the end of the “V-CDR3” AA sequence. This process was repeated for all CDR3 sequences to produce full-length V-CDR3-J AA sequences representing the full TRB variable regions from the WXS files. The software for this procedure can be found at the following link: https://github.com/arpansahoo/vdj. The full-length TRB V-CDR3-J AA sequences for both UVM and NBL can be found in Supplementary Materials Tables S1 and S2. Note, we excluded from further analyses V-CDR3-J sequences that had a single-residue “C” as their LCS for their associated V and CDR3 sequences. This filtering practice was due to an observed lack of concordance, for cases where the LCS was a “single C”, between CDR3 AA sequences identified by the authors’ algorithm/script and those identified by the imgt.org web tool that translates nucleotide sequences into V-CDR3-J AA sequences [20]. This lack of concordance was rarely seen when the LCS for the V and CDR3 obtained from the TRB recombination included at least one or two AAs following the starting C residue of the CDR3. Also, for the analyses of this report, we only considered TRB CDR3 and V-CDR3-J AA sequences derived from blood specimens and excluded such sequences from primary tumor specimens.

### 4.3. Application of Computational Prediction Models to Assess Intrinsic Disorder of the V-CDR3-J AA Sequences

We employed several tools to detect regions of intrinsic disorder in the blood-sourced TRB V-CDR3-J AA sequences. Note, the use of full-length V-CDR3-J sequences was necessary, e.g., in contrast to inputting the CDR3 AA sequences alone, because these tools typically require inputs to be relatively long AA sequences. We first used two models from the PONDR family: VSL2 (various, short-long version 2) and VL3 (various, long version 3), which were available at http://pondr.com as a web application; we designed and scripted a web crawler (Python/Selenium) to obtain VSL2 and VL3 results in a high-throughput manner. The VSL2 and VL3 models apply machine learning approaches to predict whether a region is disordered. VSL2 is a meta-predictor that combines two linear support vector machines, one trained on relatively short, disordered regions (≤30 residues) and one on long, disordered regions (>30 residues). The VSL2 model’s features include charge-to-hydrophobicity ratio, secondary structure probability scores from PSIPRED and PHDsec, and multiple sequence alignment outputs from PSI-BLAST [21]. VL3 is a feed-forward neural network trained solely on long disordered regions. VL3’s features include frequencies of certain AAs, average flexibility, and sequence complexity [22]. Additionally, we employed the IUPred2 short, IUPred2 long, and ANCHOR2 models, which are available for download at https://iupred.elte.hu/. In contrast to VSL2 and VL3 (the machine learning-based PONDR approaches), IUPred2 uses a biophysics-based model to identify regions of intrinsic disorder. At the core of IUPred2 is an energy estimation approach based on the assumption that disordered proteins result from insufficient stabilization of inter-residue interactions. IUPred2 uses a 20 × 20 energy predictor matrix to sum interaction energies between a residue and its intrachain neighbors, classifying residues with unfavorable summed interaction energies as disordered [23]. Note that while “IUPred2 long” identifies global disorder, IUPred2 short identifies shorter disordered regions [24]. In addition, ANCHOR2 uses an energy estimation approach like that of IUPred2, but it specifically identifies disordered binding regions that transition into an ordered state upon ligand binding. The ANCHOR2 model determines the likelihood of a residue being within a disordered binding site by considering three parameters: (1) the average IUPred2 score across the residue’s neighbors (does the residue belong to a disordered region?); (2) the residue’s individual IUPred2 score (is the residue unable to form sufficient favorable interactions with its neighbors?); and (3) the increase in favorable energy when the residue interacts with a hypothetical globular protein instead of its intrachain neighbors (is there a disorder-to-order transition?) [23,25]. Note that all of the five above-described approaches (VSL2, VL3, IUPred2 short, IUPred2 long, and ANCHOR2) output, for each residue in an AA sequence, the probability that the residue is within an intrinsically disordered region (or disordered binding site, in the case of ANCHOR2). To obtain a single value from each of the five intrinsic disorder assessments for each V-CDR3-J AA sequence, we averaged the probabilities over AA residues, resulting in five values (mean VSL2, mean VL3, etc.) for each V-CDR3-J AA sequence. Note, there are no composite scores in this report; each algorithm (e.g., VSL2 or VL3) is used independently, and the specific use of each algorithm is indicated and detailed in Section 2. See Supplementary Materials Tables S3 and S4 for a list of the intrinsic disorder values for each V-CDR3-J AA sequence assessed in this report.

### 4.4. Characterization of Four Physico-Chemical Parameters of CDR3 AA Sequences

We also assessed the blood-sourced TRB CDR3 AA sequences, independently of the V- and J-gene segments. However, the above intrinsic disorder assessment tools are not appropriate for such short sequences, so we instead assessed four other physico-chemical parameters representing disorder or stability: proportion of (a) disorder-promoting AA residues; (b) α-helix promoting residues; (c) β-turn promoting residues; and (d) β-sheet promoting residues. This was performed with the software at the link indicated above in this Section 4, specifically with functions from Biopython’s ProtParam module [26] and the Pappu group’s localCIDER package [27]. The proportion of disorder-promoting residues is calculated as the fraction of residues in an AA sequence that tend to promote disorder in a protein’s structure; these residues are T, A, G, R, D, H, Q, K, S, E, and P [28]. The other three parameters are defined as the fraction of residues in a sequence that tend to be enriched in an α-helix, β-turn, or β-sheet. For helix, the residues are M, A, L, E, and K [29]; for turn, the residues are N, P, G, S, and D [30]; and, for sheet, the residues are V, I, T, Y, F, W, and L [31]. See Supplementary Materials Table S5 for a list of the physico-chemical parameter values for each CDR3 AA sequence assessed in this report.

### 4.5. Survival Analysis

Clinical data for the UVM and NBL cases were obtained from cBioPortal.org [32,33]. We filtered NBL cases to those marked by the presence of *MYCN* amplification. For each cancer, we analyzed the association of various parameters representing the cases, such as the average proportion of disorder-promoting AA residues or the average ANCHOR2 score, with OS. Note, each parameter was calculated as the average of the total set of values (e.g., ANCHOR2 values) for the collection of the many blood-sourced TRB CDR3 or V-CDR3-J AA sequences obtained for any individual case. Cases were then split into two groups based on quantification of the case average for the parameter being assessed, i.e., upper vs. lower 50th percentile, and Kaplan–Meier (KM) survival analyses were performed with the Python package “lifelines” [26]. Statistical comparison of OS plots was conducted with the “lifelines” package’s implementation of the log-rank test. See Supplementary Materials Tables S6–S8 for the KM input data.

### 4.6. Multivariate Analysis

Multivariate Cox regression analyses were performed with the Statistical Product and Service Solutions (formerly, Statistical Package for the Social Sciences; IBM SPSS v29). These multivariate analyses were performed to expand upon the univariate survival analyses detailed above while accounting for clinical covariates [34,35]. All parameters used in the multivariate analyses first underwent a univariate assessment, and no parameter was used in the multivariate analyses without having a univariate result of *p* = 0.1 or less.

## Figures and Tables

**Table 1:** Multivariate analysis of UVM survival per intrinsic disorder assessment and clinical features.

*Analysis 1*			
**Covariate**	**Exp(B)**	**Significance**	**95% CI for Exp(B)**
***VSL2***	9.812	0.006	1.919–50.175
*Age at diagnosis*	1.049	0.169	0.980–1.124
*AJCC pathologic stage*	4.647	0.031	1.155–18.691
*AJCC pathologic T*	0.492	0.093	0.215–1.126
*Received treatment*	3.875	0.135	0.655–22.935
*Fraction of genome altered*	8.068	0.351	0.100–647.679
*MSIsensor score*	1.591 × 10^3^	0.072	0.511–4.951 × 10^6^
***Analysis 2***			
***IUPred2 short***	5.910	0.009	1.569–22.259
*Age at diagnosis*	1.039	0.237	0.975–1.107
*AJCC pathologic stage*	4.469	0.016	1.316–15.184
*AJCC pathologic T*	0.585	0.128	0.294–1.167
*Received treatment*	2.179	0.334	0.449–10.581
*Fraction of genome altered*	16.602	0.240	0.153–1.797 × 10^3^
*MSIsensor score*	2.573 × 10^3^	0.044	1.251–5.292 × 10^6^
***Analysis 3***			
***IUPred2 long***	9.355	0.002	2.218–39.453
*Age at diagnosis*	1.041	0.227	0.975–1.111
*AJCC pathologic stage*	5.052	0.013	1.405–18.167
*AJCC pathologic T*	0.568	0.122	0.277–1.163
*Received treatment*	2.073	0.374	0.415–10.354
*Fraction of genome altered*	21.022	0.212	0.175–2.519 × 10^3^
*MSIsensor score*	2.206 × 10^3^	0.051	0.969–5.024 × 10^6^
***Analysis 4***			
***ANCHOR2***	6.939	0.006	1.732–27.803
*Age at diagnosis*	1.044	0.184	0.980–1.112
*AJCC pathologic stage*	4.787	0.015	1.362–16.823
*AJCC pathologic T*	0.498	0.061	0.240–1.033
*Received treatment*	3.357	0.132	0.695–16.216
*Fraction of genome altered*	26.585	0.187	0.204–3.468 × 10^3^
*MSIsensor score*	879.865	0.089	0.357–2.171 × 10^6^
***Analysis 5***			
***Proportion of disorder-promoting residues***	4.652	0.029	1.166–18.562
*Age at diagnosis*	1.045	0.157	0.983–1.110
*AJCC pathologic stage*	2.268	0.149	0.747–6.889
*AJCC pathologic T*	0.697	0.265	0.369–1.316
*Received treatment*	3.179	0.138	0.691–14.623
*Fraction of genome altered*	17.480	0.239	0.149–2.054 × 10^3^
*MSIsensor score*	4.477 × 10^3^	0.030	2.213–9.059 × 10^6^

**Table 2:** Multivariate analysis of NBL survival per intrinsic disorder assessment and clinical features.

*Analysis 1*			
**Covariate**	**Exp(B)**	**Significance**	**95% CI for Exp(B)**
***ANCHOR2***	0.401	0.012	0.197–0.816
*Age at diagnosis*	0.795	0.196	0.561–1.126
*Gender*	0.602	0.194	0.280–1.294

## Data Availability

Most of the data upon which these analyses were based are available in the Supplementary Materials; additional data are publicly available at cbioportal.org.

## Supplementary Materials

The following supporting information can be downloaded at: https://www.mdpi.com/article/10.3390/ijms252111691/s1.
